# ACPA is a main risk factor for CT-proven pulmonary nodule progression in patients with rheumatoid arthritis

**DOI:** 10.1007/s10067-025-07344-9

**Published:** 2025-01-30

**Authors:** Güllü Sandal Uzun, Yasin Sarıkaya, Sevtap Arslan, Mustafa Ekici, Emine Büşra Ata, Oğuz Karcıoğlu, Emre Bilgin, Levent Kılıç, Sedat Kiraz, Ali İhsan Ertenli, Macit Arıyürek, Umut Kalyoncu

**Affiliations:** 1 Faculty of Medicine, Division of Rheumatology, Department of Internal Medicine, Hacettepe University, Ankara, Turkey; 2 Faculty of Medicine, Department of Radiology, Hacettepe University, Ankara, Turkey; 3 Faculty of Medicine, Department of Internal Medicine, Hacettepe University, Ankara, Turkey; 4 Faculty of Medicine, Department of Chest Diseases, Hacettepe University, Ankara, Turkey

**Keywords:** Anti-citrullinated protein antibodies, Disease-modifying antirheumatic drugs (DMARDs), Pulmonary nodules, Rheumatoid arthritis

## Abstract

**Objectives:**

To determine the features of rheumatoid pulmonary nodules and the factors associated with nodule progression in patients with rheumatoid arthritis.

**Methods:**

Between January 2010 and September 2018, RA patients with at least one chest computed tomography (CT) were included. Two experienced radiologists examined chest CTs. Nodules with changing dimensions on follow-up or at least two nodules with different sizes or cavitary nodules were considered rheumatoid pulmonary nodules. To identify follow-up changes in the nodules, progression was defined as the appearance of any new nodules or increase in the size of the nodules, regression was no new nodules and no increase in the size of any nodules and decrease in the size of at least one nodule, and stability was no appearance of new nodules and no change in the size of nodules and no disappearance of the nodule. We compared the demographics, comorbidities, RA-specific treatments, and nodule characteristics according to seropositivity. Factors that may be associated with RPN progression were studied.

**Results:**

A total of 204 (136 (66.7%) female) patients were included in the study. The median disease duration at baseline CT was 7.29 years (0.05–57.5). Pulmonary nodules were detected in the first CT of 21 (10.2%) patients before RA diagnosis, with a median time of 10.38 (0.46–254) months. The median number of nodules and median diameter of the dominant nodule were higher, and cavitation was more prevalent in seropositive patients. ACPA positivity was independently associated with progression (OR 3.69 (1.33–12.4), *p* = 0.03). Cs-DMARDs and b/ts-DMARDs, especially anti-TNF agents, did not affect nodule progression.

**Conclusion:**

Rheumatoid pulmonary nodules may precede RA, and seropositivity, especially ACPA, is an important independent risk factor for RPN occurrence and progression.

**Table:** 

**Key Points** • *Rheumatoid pulmonary nodules were mainly located peripherally, in the right lobe, and had a high cavitation rate.* • *ACPA positivity was found as a main effective factor in RPN progression. * • *Cs/b-DMARD treatments were not associated with RPN progression.*

## Introduction

Rheumatoid arthritis (RA) is an inflammatory disease with symmetrical polyarthritis, especially involving small joints. Another essential feature of the disease is that it progresses with extra-articular involvement and lung involvement is an important cause of morbidity and mortality in patients with RA [1, 2]. Rheumatoid pulmonary nodule (RPN) is usually asymptomatic, and its prevalence rate is as low as 0.4% in routine chest X-rays; it rises to 33% with computed tomography (CT) scans and lung biopsies [3–5].

Rheumatoid pulmonary nodules can be detected on X-ray or CT scan during evaluation for infection, unexplained pulmonary symptoms, acute phase elevation, malignancy screening, or incidentally. Although lung biopsy is the gold standard for distinguishing benign and malignant nodules, it is unfeasible to perform it on every RA patient due to the invasive of the procedure. It is crucial to differentiate RPNs from malign nodules. Although it is not conducted for RPNs specifically, The Fleischner Society Guidelines helps us to predict the nature of nodules without using invasive procedures [6]. A recent study published on the differentiation of pulmonary rheumatoid and malignant nodules. They argued that ≥ four nodules, peripheral localization, cavitation, satellite nodules, sharp borders, and subpleural rind were found to have an optimal sensitivity of 77% and a specificity of 92% for RPN [6, 7].

RA-related factors affect the frequency of occurrence of RPN: seropositivity, male gender, age, disease duration, and subcutaneous nodules [2]. Studies have shown that bDMARDs (biological disease-modifying antirheumatic drugs), such as rituximab, jak inhibitors, and tocilizumab reduce nodule size [8, 9]. On the other hand, csDMARDs (conventional synthetic disease-modifying antirheumatic drugs), such as methotrexate (MTX) and leflunomide, and bDMARDs such as anti-TNF agents have been accused of nodule development [10, 11]. Although male gender, smoking, and seropositivity are associated with nodule development, the literature on the factors affecting the change of pulmonary rheumatoid nodule size mainly focused on RA-specific treatments [12]. Consequently, there is limited literature on factors affecting nodule size (such as characteristic features of the nodule and disease-related factors) apart from treatment.

Our study aimed to examine RPN patients followed up in our clinic in terms of nodule characteristics, progression, and all factors associated with nodule progression.

## Methods

### Patients selection and study design

**Fig. 1:**
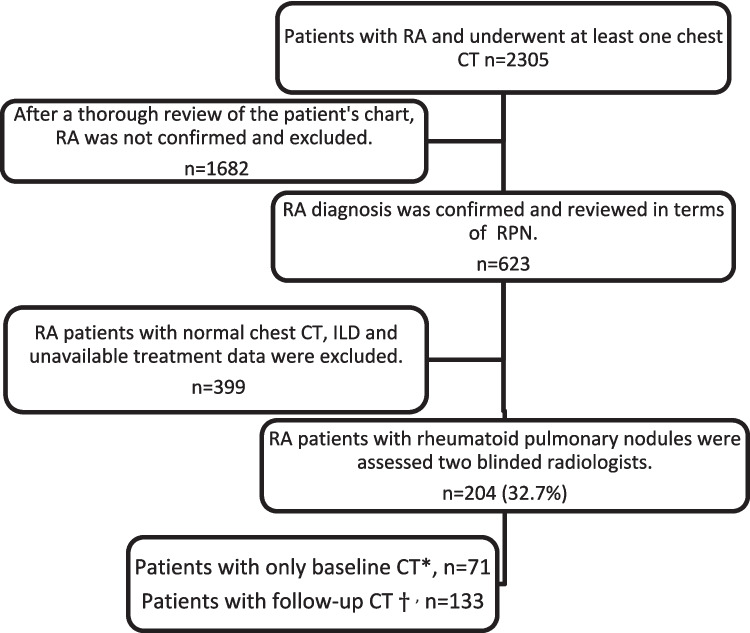
Selection of RA patients with rheumatoid pulmonary nodules. *The first CT in which the nodule was detected was defined as “baseline CT.” ^†^The last CT with at least two CTs and nodule change was described as “follow-up CT.” Abbreviations: CT, computed tomography; ILD, interstitial lung disease; RA, rheumatoid arthritis

Patients who applied to the Hacettepe University rheumatology clinic between January 2010 and September 2018 and had a chest CT scan at least once were screened for the study. The current study is a retrospective observational study. We further screened for the ICD-10 codes for RA (M05 and M06) from the hospital’s electronic files and patients’ charts [13]. Selection of patients with rheumatoid lung nodules is given in Fig. 1. A total of 623 patients had a confirmed RA diagnosis according to the 2010 American College of Rheumatology (ACR)/European League Against Rheumatism (EULAR) classification criteria [14]. The first CT in which the nodule was detected was defined as “baseline CT.” The last CT with at least two CTs and nodule change was described as “follow-up CT.” Patients with follow-up CT were further analyzed and divided into three groups according to “progressed,” “regressed,” or “stable” according to the change in RPN size described as follows.

Demographic and disease-specific variables were recorded. The patients were also examined regarding bronchoalveolar lavage (BAL) and histopathology. Patients with pulmonary nodules and whose histopathology revealed malignancy, bacterial and fungal infection, or tuberculosis were excluded from the study. Treatments were recorded as ever exposure to glucocorticoids, csDMARDs (MTX, leflunomide, sulfasalazine, hydroxychloroquine, bDMARDs (antitumor necrosis factor (anti-TNF) drugs including etanercept, infliximab, adalimumab, golimumab, certolizumab), rituximab, abatacept, tocilizumab), and targeted synthetic DMARD (tofacitinib, baricitinib). The treatments were also documented between the baseline and follow-up CT to examine for effect on progression. Rheumatoid factor (RF) (< 20 IU/mL) and anti-citrullinated protein/peptide antibody (ACPA) (0–5 RU/mL) were classified as normal. If there is an RF of ACPA positivity above these values, it is recorded “seropositive.” Otherwise, it is called “seronegative.” Additionally, “low-level seropositivity” up to three times the normal upper level and “high-level seropositivity” over the three times upper normal level [14]. Patients with RPN were divided into two groups according to seropositivity.

### Radiological assessment

Reconstructed CT images with a slice thickness of 1 mm, 1.25, or 1.5 mm performed on different CT scanners (Somatom Force, Siemens Healthineers, Erlangen, Germany; Optima CT540, GE Healthcare, Chicago, IL; Sensation 16, Siemens Healthineers, Erlangen, Germany) were evaluated by two radiologists with 5 and 7 years of thoracic radiology experience. These two radiologists were blinded to the patients’ clinics and each other. For each patient, the number of nodules and nodule characteristics (size, cavitation, calcification, and location (left or right lung, upper/middle/lower zone, and peripheral/central/subpleural)) were determined in the axial plane. Zones were determined according to the following criteria: upper zones were above the level of the carina, middle zones were between the level of the carina and lower border of inferior pulmonary veins, and lower zones were below the lower border of inferior pulmonary veins [15]. Nodules abutting the pleural surfaces directly were named subpleural nodules. Nodules around peripheral pulmonary arterial branches or 3–5 mm away from the pleura, interlobular septa, or pulmonary veins were categorized as central nodules [16]. The rest of the nodules were determined as peripheral nodules. Changing the dimension, cavitation, and calcification of nodules were evaluated on follow-up CTs. New pulmonary nodules on follow-up CTs were noted. The presence of pleural effusion and pleural thickening were also investigated.

### Description of the rheumatoid nodule and its progression

i. **Inclusion criteria for rheumatoid pulmonary nodule **[2, 17, 18]
  1. Nodules with size changes on follow-up
  2. At least two nodules of different sizes. We added this criterion to increase the diagnostic accuracy of a rheumatoid pulmonary nodule.
  3. Nodule with any cavitation on chest CT
ii. **Exclusion criteria**
  1. A nodule size of less than 5 mm. In the nodule follow-up guide, nodules below 5 mm are seen in the average population, with no follow-up indication. Therefore, smaller than 5 mm nodules are not included to increase the diagnostic accuracy of the RPN [6, 7].
iii. **Description progression, regression, or stable-sized nodules**

Response Evaluation Criteria in Solid Tumors (RECIST 1.1) classification was used to assess rheumatoid pulmonary nodule progression status [9]. However, this system was developed to evaluate malignant nodules [6, 19, 20]. Since the rheumatoid pulmonary nodules are benign, we used an arbitrary algorithm based on the clinical and radiological expertise of the authors. Cavitation and calcification were considered separate entities.

The following criteria were used to determine the “progressed,” “regressed,” or “stable” nodules:

1. Progression: Appearance of any new nodules OR increase in the size of the existing nodules
2. Regression: No new nodules AND no increase in the size of any existing nodules and decrease in the size of at least one existing nodules or decrease in size/disapperance of a nodule.
3. Stable: No new nodules AND no change in the size of existing nodules AND no disappearance of the existing nodule

### Ethical approval

The study was approved by the Ethics Committee of Hacettepe University Faculty of Medicine (No. GO: 18–957; date: 17 November 2019) and was conducted in accordance with the Declaration of Helsinki of 1975/83.

### Statistical analyses

Statistical analysis was performed using SPSS version 26.0 (SPSS Inc., Chicago, IL). The variables were calculated using visual (histogram, normality plots) and analytic methods (Kolmogorov–Smirnov) to determine whether or not they were normally distributed. The descriptive analysis was determined using mean ± standard deviation (SD) or the median, interquartile range (IQR). Chi-square was used to compare categorical variables. Student’s *t*-test and Mann–Whitney *U* test were used to compare the two groups’ normally and nonnormally distributed continuous data. One-way ANOVA and subsequent post hoc tests were used to compare rheumatoid pulmonary nodule groups at the follow-up CT. Factors associated with the nodule progression were identified by univariate analysis (*p* < 0.20) and further entered into logistic regression by backward LR to determine independent predictors of progression. A *p*-value of < 0.05 was accepted as statistically significant.

## Results

### Characteristics of patients with rheumatoid pulmonary nodules

Of the 204 patients included in the study, 136 (66.7%) were female, and the median disease duration at baseline CT was 7.29 years (0.05–57.5). The number of patients with rheumatoid pulmonary nodules (RPNs) detected on baseline chest CT was 204 (32.7%). A total of 133 (65.1%) of these patients had follow-up CTs. BAL was performed in 18 (8.8) patients and 16 (7.8) of them were histopathologically diagnosed as RPN. Of these 204 patients, 155 (75.9%) were seropositive. In seropositive patients, 135 (88.2) were RF positive, and the mean RF titer was 381 (± 594) IU/mL. One hundred twelve (72.2%) of the RF-positive patients had a high level RF. In seropositive patients, 102/122 (83.6%) was ACPA positive, and the mean ACPA titer was 445 (± 622) RU/mL. A high level ACPA positivity was found in 94/119 (78.9%) ACPA-positive patients.

The most common comorbid diseases were hypertension 87/158 (55%), hyperlipidemia 43/156 (27.5%), and chronic obstructive lung disease 26/157 (22.9%). The prevalence of comorbid diseases did not differ significantly between seropositive and seronegative patients.

Nodules were detected in the first CT of 21 (10.2%) patients before RA diagnosis. Time to RA diagnosis with baseline CT was a median time of 10.3 (0.46–254) months. Of these 21 patients, 10 patients underwent CT for malignancy screening, 3 for pulmonary thromboembolism, 4 for infection, and 3 for unknown reasons.

The treatment before baseline CT was documented. These were *n* = 96 (48.5%) glucocorticoids, 47 (23.7%) sulfasalazine, 85 (42.9%) hydroxychloroquine, 62 (31.3%) leflunomide, 72 (36.3%) MTX, 26 (13%) anti-TNF agents, 9 (4.5%) rituximab, 4 (2%) abatacept, 1 (0.5%) tofacitinib, and 1 (0.5%) tocilizumab.

### The characteristic features of rheumatoid pulmonary nodules

**Fig. 2:**
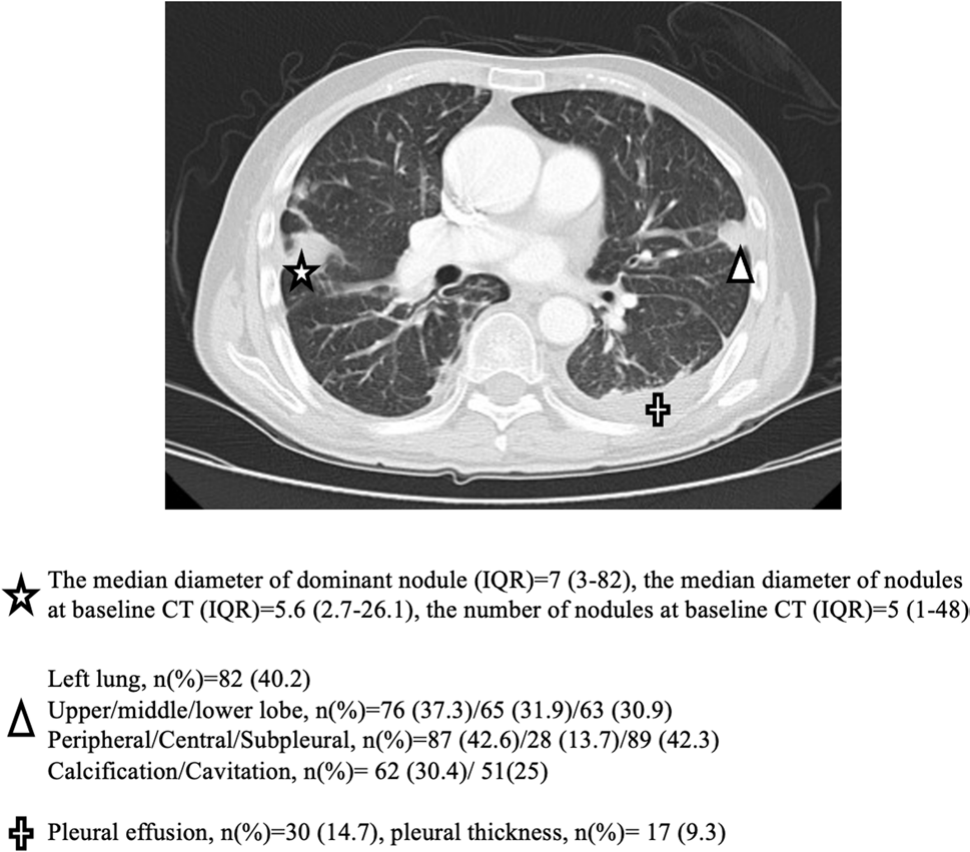
The localization and characteristics of rheumatoid pulmonary nodules

The localization characteristic features of rheumatoid pulmonary nodules are summarized in Fig. 2. The median number of nodules and median diameter of the dominant nodule were higher, and cavitation was more prevalent in seropositive patients (Table 1). Exposure to leflunomide and rituximab is more common in the seropositive group.

**Table 1:** Demographic and clinical characteristics of all RPN patients and comparison by seropositivity

Characteristics	All patients *n* = 204	Seropositive *n* = 155 (76.0%)	Seronegative *n* = 49 (24.0%)	*p*
**Age at the baseline CT, mean (± SD)**	58.5 (12.7)	59.0 (10.1)	57.2 (15.6)	0.47
**Female, *n* (%)**	136 (66.7)	99 (64.7)	37 (72.5)	0.2
**Smoking, *n* = 76 (ever)**	43 (56.5)	34 (55.7)	9 (60)	0.36
**Cigarette pack/year, (med-IQR)**	25 (1 − 120)	25 (1 − 120)	22.5 (4 − 40)	0.61
**Disease duration, (med, IQR)**	12.9 (0.1 − 61.2)	13.0 (0.1 − 61.4)	12.2 (0.2 − 38.5)	0.86
**RA-specific treatments (ever),** ***n*** **(%)**				
• **Methotrexate, *n* = 177** • **Leflunomide, *n* = 185** • **Sulfasalazine, *n* = 178** • **Hydroxychloroquine *n* = 176** • **Anti-TNF agents, *n* = 167** • **Rituximab, *n* = 168** • **Abatacept, *n* = 164** • **Jak inhibitors *n* = 179** • **Tocilizumab, *n* = 173**	102 (57.6) 114 (62.6) 73 (41) 136 (77.2) 45 (26.9) 39 (23.2) 14 (8.5) 14 (7.8) 10 (5.7)	85 (61.2) 99 (68.8) 59 (42.1) 108 (79.4) 38 (29.5) 36 (27.7) 11 (8.6) 12 (8.7) 8 (5.9)	17 (44.7) 15 (36.6) 14 (36.8) 28 (70) 7 (18.4) 3 (7.9) 3 (8.3) 2 (4.9) 2 (5.3)	0.052 < 0.001 0.3 0.1 0.1 0.007 0.6 0.3 0.6
**Pleural effusion, *n* (%)**	30 (14.7)	22 (14.2)	8 (16.3)	0.4
**Pleural thickness, *n* (%)**	17 (9.3)	11 (7.7)	6 (15)	0.3
**Number of nodules in baseline CT, (med-IQR)**	5 (1 − 48)	5 (1 − 48)	3 (1 − 27)	0.003
**Diameter of nodules at baseline CT, (med-IQR)**	5.6 (2.7 − 26.1)	5.6 (2.7 − 26.1)	5.5 (3 − 20)	0.52
**Diameter of dominant nodule, (med-IQR)**	7 (3 − 82)	8 (3 − 82)	6 (3 − 45)	0.01
**Localization of dominant nodule**				
• **Right** • **Upper** • **Middle** • **Lower** • **Peripheral** • **Central** • **Subpleural**	121 (59.3) 76 (37.3) 65 (31.9) 63 (30.9) 87 (42.6) 28 (13.7) 89 (43.6)	98 (61.9) 61 (39.4) 47 (30.3) 47 (30.3) 61 (39.4) 25 (16.1) 69 (44.5)	25 (52.9) 15 (30.6) 18 (36.7) 16 (32.7) 26 (53.1) 3 (6.1) 20 (40.8)	0.1 0.5 0.08
**Cavitation, *n* (%)**	51 (25)	44 (28.4)	7 (14.3)	0.032
**Calcification, *n* (%)**	62 (30.4)	47 (30.3)	15 (30.6)	0.5

*SD* standard deviation, *IQR* interquantile range, *RF* rheumatoid factor, *ACPA* anti-citrullinated protein antibodies, *CT* computed tomography, *Anti-TNF* anti-tumor necrosis factor inhibitor

### Characteristics of patients with progression on follow-up CT

One hundred thirty-three out of 204 (65.2%) patients had at least two CT scans during follow-up. The median time between the baseline and follow-up CT was 25.5 months (2.4–157.6). During this period, 39 (29.3%) patients had progression, 27 (20.3%) patients had regression, and 67 (50.3%) patients remained stable. For ACPA positivity and cavitation rate, significant differences (all *p* < 0.05) were detected (a) between the 3 RPN groups using the one-way ANOVA. ACPA positivity was more frequent in the progressed group compared to the stable group (*p*2 = 0.03). Moreover, cavitation was significantly frequent in the progressed group compared to the stable group (*p*2 =  < 0.001). There was no difference between the groups regarding the nodule size, location, and calcification features. All group characteristics are given in Table 2.

**Table 2:** Characteristics of patients with changing on follow-up CT

	**Regression** ***N* = 27** **(Group 1)**	**Progression** ***N* = 39** **(Group2)**	**Stable** ***N* = 67** **(Group 3)**	***p***	***p*1**	***p*2**	***p*3**
**Female, *n* (%)**	14 (51.9)	26 (66.7)	47 (70.1)	0.2	NA	NA	NA
**Age at the time of CT, mean (± SD)**	55.9 (12)	60.4 (11.4)	56.8 (13.1)	0.56	NA	NA	NA
**Disease duration (med-IQR)**	12.4 (0.2–40.1)	11.6 (2.2–32.8)	13 (0.2–41.2)	0.9	NA	NA	NA
**Duration until to follow up CT, years (med-IQR)**	1.11 (0.08–33.81)	1.67 (0.08–9.5)	2.4 (0.08–31)	0.18	NA	NA	NA
**RF positive (reference range < 20 IU/mL) *n* (%)**	18 (66.7)	27 (69.2)	40 (59)	0.3	NA	NA	NA
**RF titer IU/mL (med-IQR)**	313 (38–4040)	136 (0–1230)	171 (0–2870)	0.087	NA	NA	NA
**RF titer, over 3x, *n* (%)**	20 (74.1)	21 (53.8)	37 (55.2)	0.2	NA	NA	NA
**ACPA positive, (reference range 0–5 RU/mL) *n* (%)**	15/23 (65.2)	26/30 (86.6)	28/49 (57.1)	0.03	0.06	0.38	0.007
**ACPA titer, over 3x, *n* (%)**	14/21 (66.6)	25/30 (83.3)	25/44 (56.8)	0.6	NA	NA	NA
**ACPA titer RU/mL (med-IQR)**	250 (0–3200)	222 (0–320)	250 (0–1000)	0.56	NA	NA	NA
**RF or ACPA positive, *n* (%)**	24 (88.9)	33 (84.6)	46 (68.7)	0.04	0.45	0.03	0.054
**Smoking, *n* = 76 (ever)**	11/14 (78.5)	11/20 (55)	11/25 (44)	0.1	NA	NA	NA
**Pleural effusion, *n* (%)**	6 (22.2)	8 (20.5)	4 (6)	0.08	NA	NA	NA
**Pleural thickness, *n* (%)**	4 (14.8)	3 (7.7)	4 (6)	0.6	NA	NA	NA
**Localization of dominant nodule,** ***n*** **(%)**							
• **Right** • **Left** • **Upper** • **Middle** • **Lower** • **Peripheral** • **Central** • **Subpleural**	13 (48.1) 14(51.9) 12 (44.4) 5 (18.5) 10 (37) 15 (55.6) 1 (3.7) 11(40.7)	22 (56.4) 17(43.6) 13 (33.3) 8 (20.5) 18 (46.2) 15 (38.5) 5 (12.8) 19 (48.7)	42 (62.7) 25(37.3) 24 (38.5) 28 (41.8) 15 (22.4) 24 (35.8) 15 (22.4) 28 (41.8)	0.4 0.4 0.6 0.022 0.035 0.2 0.06 0.7	NA NA NA 0.55 0.3 NA NA NA	NA NA NA 0.026 0.17 NA NA NA	NA NA NA 0.020 0.01 NA NA NA
**Cavitation, *n* (%)**	12 (44.4)	20 (51.3)	9 (13.4)	< 0.001	0.46	0.002	< 0.001
**Calcification, *n* (%)**	7 (25.9)	13 (33.6)	19 (28.4)	0.7	NA	NA	NA
**Number of nodules in baseline CT (med-IQR)**	5 (2–24)	5 (1–36)	3 (1–27)	0.20	NA	NA	NA
**Diameter of nodules at baseline CT (med-IQR)**	5.5 (3.3–16)	6.8 (3–26.1)	5 (3–15)	0.22	NA	NA	NA
**Diameter of dominant nodule (med-IQR)**	8 (4–40)	9 (3–82)	7 (3–30)	0.24	NA	NA	NA
**RA-specific treatments at between baseline CT and follow-up CT,** ***n*** **(%)﻿**							
• **Glucocorticoids** • **Methotrexate** • **Leflunomide** • **Sulfasalazine** • **Hydroxychloroquine** • **Anti-TNF agents** • **Rituximab** • **Abatacept** • **Jak-inhibitors** • **Tocilizumab**	18 (66.7) 9 (33.3) 15 (55.6) 7 (25.9) 15 (55.6) 2 (7.4) 3 (11.1) 3 (11.1) 1 (3.7) 0	22 (56.4) 13 (33.3) 27 (69.2) 14 (35.9) 23 (59) 8 (20.5) 5 (12.8) 1 (2.6) 2 (5.1) 1 (2.6)	43 (64.2) 29 (43.3) 39 (58.2) 21 (31.3) 47 (70.1) 10 (14.9) 6 (9) 0 1 (1.5) 0	0.73 0.49 0.43 0.6 0.30 0.1 0.8 0.3 0.01 0.05 0.3	NA 0.18 NA NA	NA 0.022 NA NA	NA 0.36 NA NA

Data were analyzed through one-way ANOVA followed by Tukey’s post hoc test for independent variables. Statistically significant differences (*P* < 0.05) are shown in bold

*SD* standard deviation, *IQR* interquantile range, *NA* not applicable, *RF* rheumatoid factor, *ACPA* anti-citrullinated protein antibodies, *CT* computed tomography, *Anti-TNF* anti-tumor necrosis factor inhibitors

*p*1 = Comparison of regression and progression groups

*p*2 = Comparison of regression and stable groups

*p*3 = Comparison of progression and stable groups

When we compare the nodule characteristics between regressed (27 (20.3%)) and non-regressed (106 (79.6%)) groups, regressed nodules were predominantly located peripherally (*p* = 0.02). There was no difference in other parameters between these two groups.

The patients were divided into two groups with progression (*n* = 39), no progression (*n* = 94), and factors associated with progression were studied. Progression was more frequent in ACPA-positive patients (log-rank *p* = 0.03). The odds ratio of the mean nodule diameter was close to the significance limit (log-rank *p* = 0.07). The utilization of b/ts-DMARDs, anti-TNF agents, MTX, hydroxychloroquine, leflunomide, and glucocorticoids did not demonstrate a statistically significant impact on the progression of nodules. The factors associated with progression are shown in Table 3.

**Table 3:** Univariable and multivariable analysis for factors associated with progression of rheumatoid pulmonary nodule

	Univariable analysis		Multivariable analysis	
	Adjusted odds ratio (95% CI)	*p*	Adjusted odds ratio (95% CI)	*p*
ACPA positivity, *n* (%)	4.23 (1.33–13.4)	0.01	3.69 (1.33–12.4)	0.03
Diameter of nodules at baseline CT (med-IQR)	1.11 (1.0–1.2)	0.04	1.15 (0.98–1.36)	0.072
Diameter of dominant nodule, (med-IQR)	1.03 (1.0–1.07)	0.05	1.09 (0.85–1.4)	0.42
Cavitation, *n* (%)	3.3 (1.49–7.30)	0.003	1.50 (0.55–4.3)	0.33
Duration until to follow up CT, years (med-IQR)	0.99 (0.98–1.00)	0.16	0.9 (0.99–1.08)	0.81
Pleural effusion, *n* (%)	1.65 (0.65–4.2)	0.11	1.38 (0.38–4.9)	0.65
RF titer IU/mL (med-IQR)	0.82 (0.53–1.27)	0.18	1.42 (0.86–3.9)	0.44
B/ts-DMARD + csDMARD combined usage, *n* (%)	0.41 (0.18–0.92)	0.03	0.97 (0.27–3.3)	0.96
Anti-TNF agents, *n* (%)	0.56 (0.21–1.51)	0.15	0.58 (0.18–1.7)	0.35
Methotrexate (ever), *n* (%)*	1.35 (0.62–2.96)	0.44		
Methotrexate < 15 mg, *n* (%)	1.28 (0.32–3.45)	0.36		
Methotrexate ≥ 15 mg, *n* (%)	1.1 (0.46–1.38)	0.52		
Leflunomide, *n* (%)	1.66 (0.75–3.68)	0.21		
Hydroxychloroquine, *n* (%)	1.34 (0.62–2.90)	0.44		
Glucocorticoids, *n* (%)	0.66 (0.31–1.43)	0.30		

*SD* standard deviation, *IQR* interquantile range, *RF* rheumatoid factor, *ACPA* anti-citrullinated protein antibodies, *CT* computed tomography, *CI* confidence intervals, *Anti-TNF* anti-tumor necrosis factor inhibitors, *csDMARD* conventional synthetic disease-modifying anti-rheumatic drugs, *bDMARD* biological disease-modifying anti-rheumatic drugs

In our study, 96 (47%) patients used MTX until baseline CT. MTX treatment was discontinued in 23 (11.2%) patients with RPN during follow-up. There was no difference in progression between patients whose MTX treatment was discontinued and those who did not (*p* = 0.1).

## Discussion

We described the features of rheumatoid pulmonary nodules detected on CT in RA patients. Accordingly, approximately 10% of patients had rheumatoid nodules before the diagnosis of RA. The nodules were evenly distributed in the lobes of the lung. Nodules are generally located in the subpleural and peripheral regions. The median number of nodules is 5. While median diameter of the dominant nodule is 7 mm; it has been observed to reach up to 8 cm in diameter. Cavitation was observed in one out of four patients. In RA patients with follow-up CT, after approximately 1.5 years, nodules remained stable in half of the patients, whereas nodule progression was detected in about 30% and regression in 20%. RA-specific treatments did not affect nodule progression. ACPA positivity was the most important risk factor for nodule progression.

The incidence of pulmonary nodules on CT scans performed for other reasons varies, but exceeds 20% [5]. In a recent study, the frequency of pulmonary nodules was 24–31% in 425,581 chest CT imaging performed on more than 200,000 adults [21]. In two retrospective studies, lung nodules detected in multislice coronary CT angiography due to coronary artery disease were studied, and their frequency was determined as 36.4% and 48.4% [22, 23]. In two recent studies on RPN, the nodule-malignancy relationship and the effect of treatments on nodule size were investigated. However, no further comments were made regarding the frequency of RPN in these studies [7, 9]. In the current study, the frequency of nodules was found to be 32.7% in patients with RA who had a CT scan at least once for any reason.

Interestingly, in 21 (10.2%) of patients in the current study, RPNs preceded the RA diagnosis, slightly higher than the literature [24]. Even though this discordance may be due to selection bias, patients with pulmonary nodules without any RA clinic should be followed carefully regarding inflammatory diseases such as rheumatoid arthritis, especially in the first year.

The nodule characteristics such as size, location, and density should be considered for the differential diagnosis of the nodule’s nature when the biopsy was not performed [25]. In a 10-year follow-up study of incidentally detected lung nodules, 60% (36–50%) of the nodules were located in the right lobe, and 57% were multiple. The distribution of the upper and lower lobes in the right and left lobes was similar (22–24%) [26]. In our study, all RPNs were multiple, and the nodules were primarily located in the right lobe (59.3%). The right middle lobe involvement was 31.9% higher than the literature data regarding the general nodules but similar to rheumatoid pulmonary nodules. Koslow et al. reported nodules located in the middle lobe of 34% of patients [7].

Regarding the location, our findings parallel the literature on general or rheumatoid pulmonary nodules [7, 27]. Although the total number of rheumatoid pulmonary nodules was similar to current literature, the median size of the dominant nodule was slightly lower in our study, which may be due to our strict inclusion criteria for classifying a nodule as a rheumatoid nodule [7, 28]. Cavitation identified in 25% of nodules in our study, much higher than the rate observed in incidental lung nodules (7%) [29]. Koslow et al. reported that about 40% of the RPNs in their study had cavitation in their study [7]. We also showed that cavitation was related to seropositivity and nodule progression. With all these findings, we can speculate that cavitation is an important and discriminating factor for RPNs. As a result, when we compare all nodule features with the literature, we considered that subpleural-located, multiple, and cavitating nodules might support the diagnosis of RPN.

In this study, after a median of 25.5 (2.4–157.6) months, 50.3% of RPN progressed, 29.3% was stable, and 20.3% was regressed, which is mainly different from incidentally detected pulmonary nodules, possibly due to the different nature of RA. Carol et al. reported were 82% stable, 12% regressed, and 6% progressed after a median of 170 (0–1574) days [30]. One of the main differences between our study and the current literature was the approximately three times longer follow-up duration. It may be necessary to follow up for a sufficient time before commenting on the course of the nodule.

ACPA positivity had the highest odds ratio for nodule progression (OR 3.69 (1.33–12.4); 95% CI). Seropositivity has long been known to increase the risk of RA and lung involvement [31, 32]. It has been shown that increased citrated proteins in the lung cause the development of RA by causing proinflammatory cytokine release and immune activation [33]. Although the pathogenetic effect of ACPA on interstitial lung disease is well known, its impact on rheumatoid pulmonary nodules is still limited. In a cohort of patients with early RA, it was shown that 34% of patients with early RA and ACPA positivity developed RPN. This study showed ACPA positivity as an independent risk factor for developing parenchymal lung disease (OR 3.9 (3.2–4.5); 95% CI) [34, 35]. It is emphasized that nodule progression is more common in seropositive patients, but it has not been stated how much there is a-fold risk. ACPA-positive RA patients should be closely monitored for nodule progression in daily rheumatology outpatient clinics.

Another factor known to affect RPN progression is the agents used to treat RA. In our study, it was shown that csDMARD and b/ts-DMARD therapy was not associated with nodule progression. However, it is well known that MTX, one of the RA-specific treatments, causes accelerated nodulose and pneumonitis, and case studies show that it causes progression in RPN [36, 37]. In our study, 47% of the patients with RPN nodules on the first CT were using MTX. During the follow-up period, MTX treatment was discontinued in 23 (11.2%) patients who developed RPN. There was no difference in progression between patients who stopped and did not discontinue MTX therapy. Another hesitation is for leflunomide (LEF), one of the cornerstones of RA treatment. In a case published by Yoshikawa, a patient developed a pulmonary nodule while using leflunomide treatment, and RPN regressed after treatment was discontinued [10]. No relationship was found between the usage of LEF and progression in the period until the follow-up-CT in our study. The impact of biological therapies on RPN is complex. Studies report that using anti-TNF agents causes RPN and that RPN-related symptoms regress after drug withdrawal [38]. Case reports of the positive effect of jak-inhibitors on RPN have been published before. A recent study by Karadeniz et al. found no relation between bDMARDs and RPN progression. However, a regression was found in the nodule size of patients using tofacitinib [9]. According to the results of the French RTX registry study, it was observed that RPN nodule size did not regress, and new nodule formation did not occur 12.8 months after switching to the rituximab (RTX) treatment [39]. In our study, the number of patients who received RTX therapy needs to be increased to make further comments. However, our study showed that all bDMARDs did not cause nodule progression.

Our study showed that nodule characteristics did not affect nodule progression (log-rank *p* = 0.072). However, the effect of mean nodule size on nodule progression was found to be close to the limit of significance. It was shown that dimension > 10 mm, part-solid or subsolid components, and spicular or lobular shape were found to be associated with an increased risk of malignancy [40]. Our study showed that the median nodule diameter did not affect progression and regression.

The main limitation of our study is its retrospective design. The lack of standardization of treatments applied in daily rheumatology practice and the low number of patients using bDMARDs may affect the results. The results may have been affected due to the difference in the chest CT imaging procedure. We have to underline that our results can not be generalized to all RA patients as we selected a particular group of RA patients. The study did not include disease activity scores due to missing data in the laboratory and physical examination findings. Nodule diagnosis confirmation with biopsy in RPN patients could not be made in all patients. On the other hand, our study has the most significant number of patients in the literature on rheumatoid pulmonary nodules. Two radiologists re-evaluated the CTs of the patients included in the analysis, and RPN progression was studied in patients with follow-up CT. The review of nodule change in 133 patients’ follow-up CT made it possible to study the factors predicting progression.

In conclusion, the frequency of RPN is 32.7% in patients diagnosed with RA and undergoing CT for any reason. In our cohort, ACPA positivity is the strongest factor associated with RPN progression in follow-up CT. The progression was relatively high in the group with a high mean nodule diameter. On the other hand, the usage of b/tsDMARDs or csDMARDs did not affect RPN progression. Our outcome data will guide the follow-up of a patient with RA and pulmonary nodules in daily rheumatology practice.

## Data Availability

Data are available upon a reasonable request.
